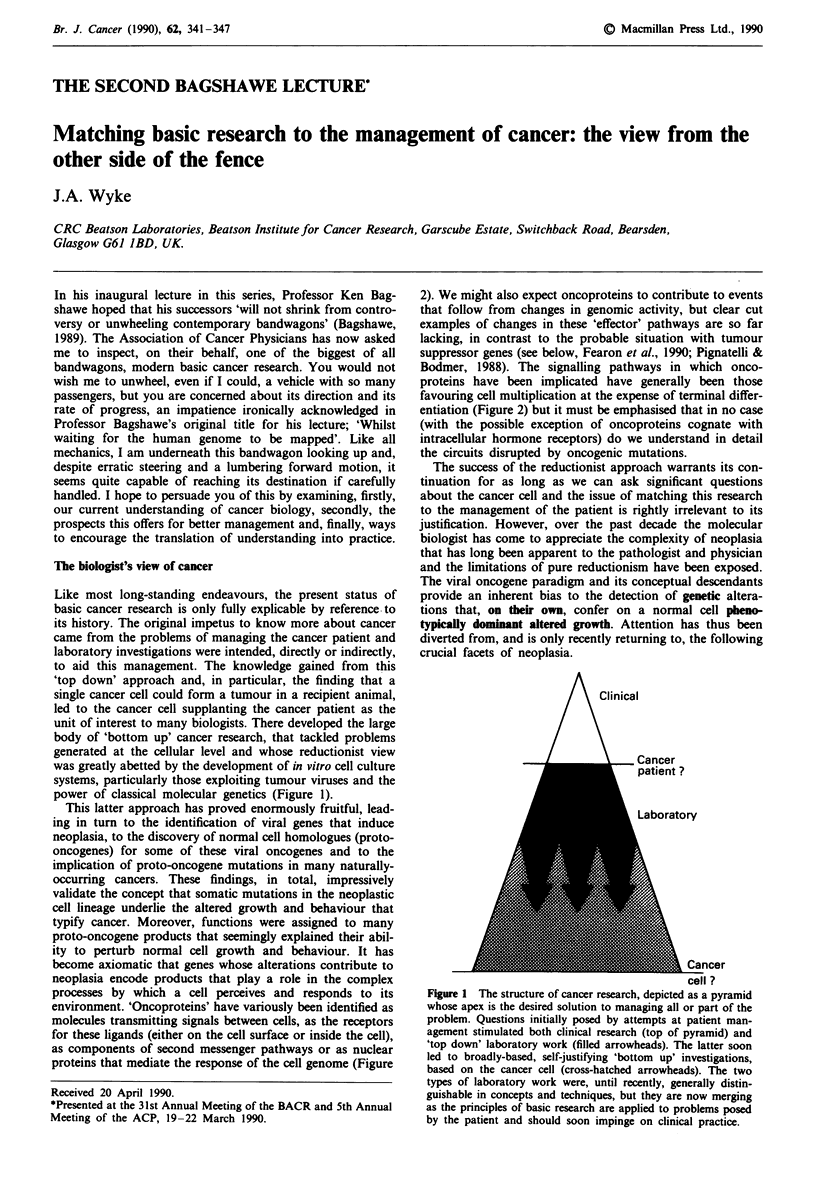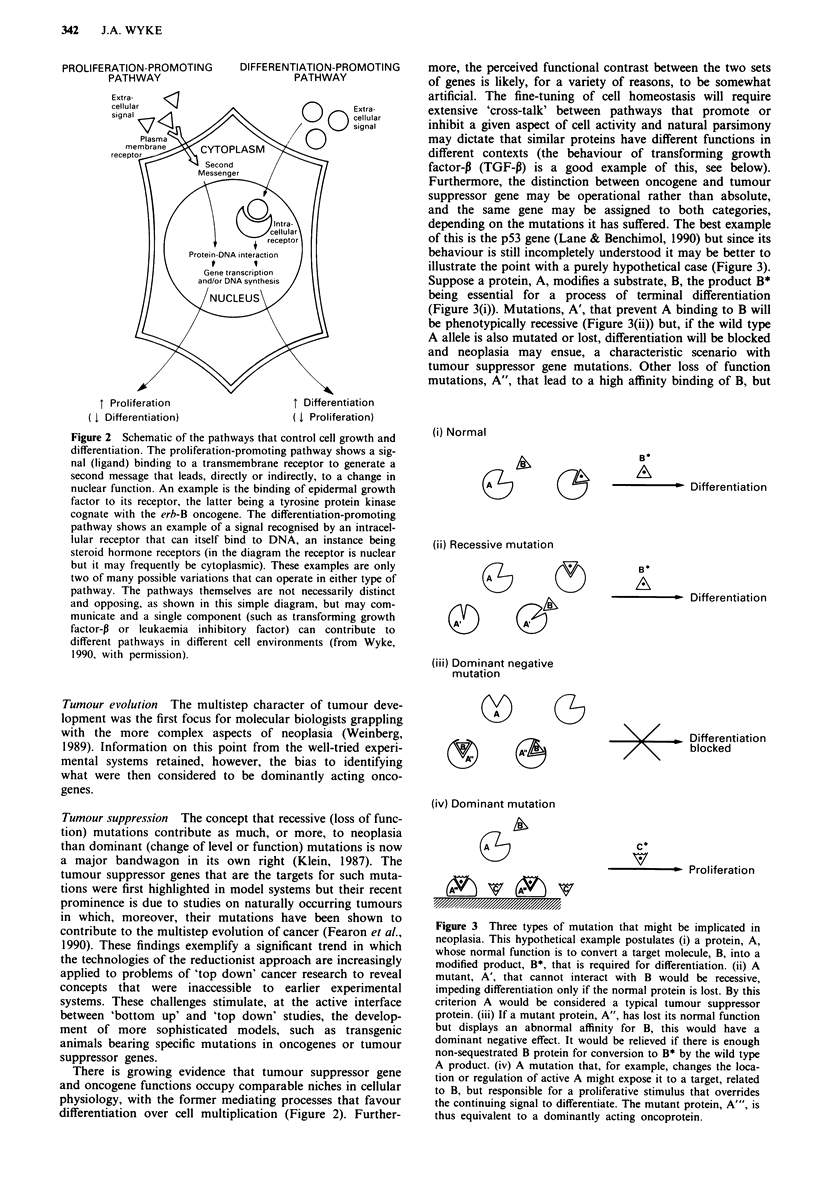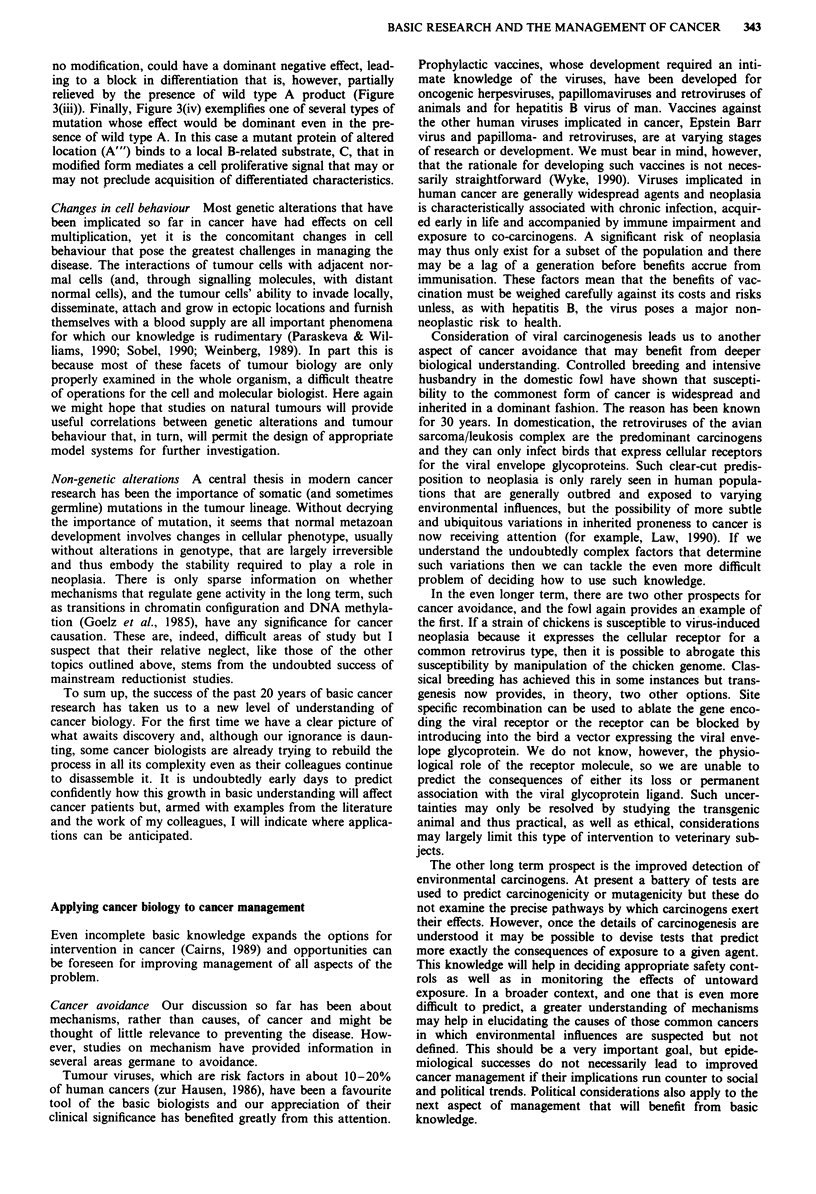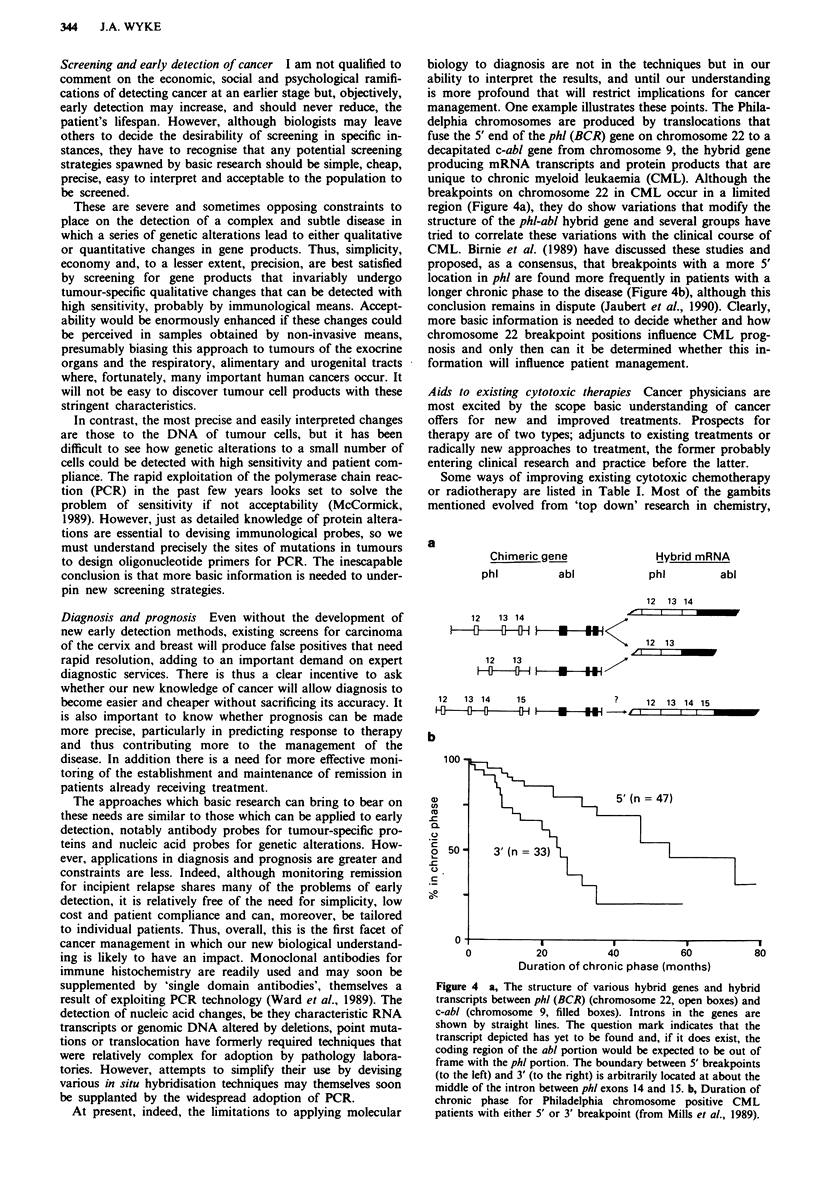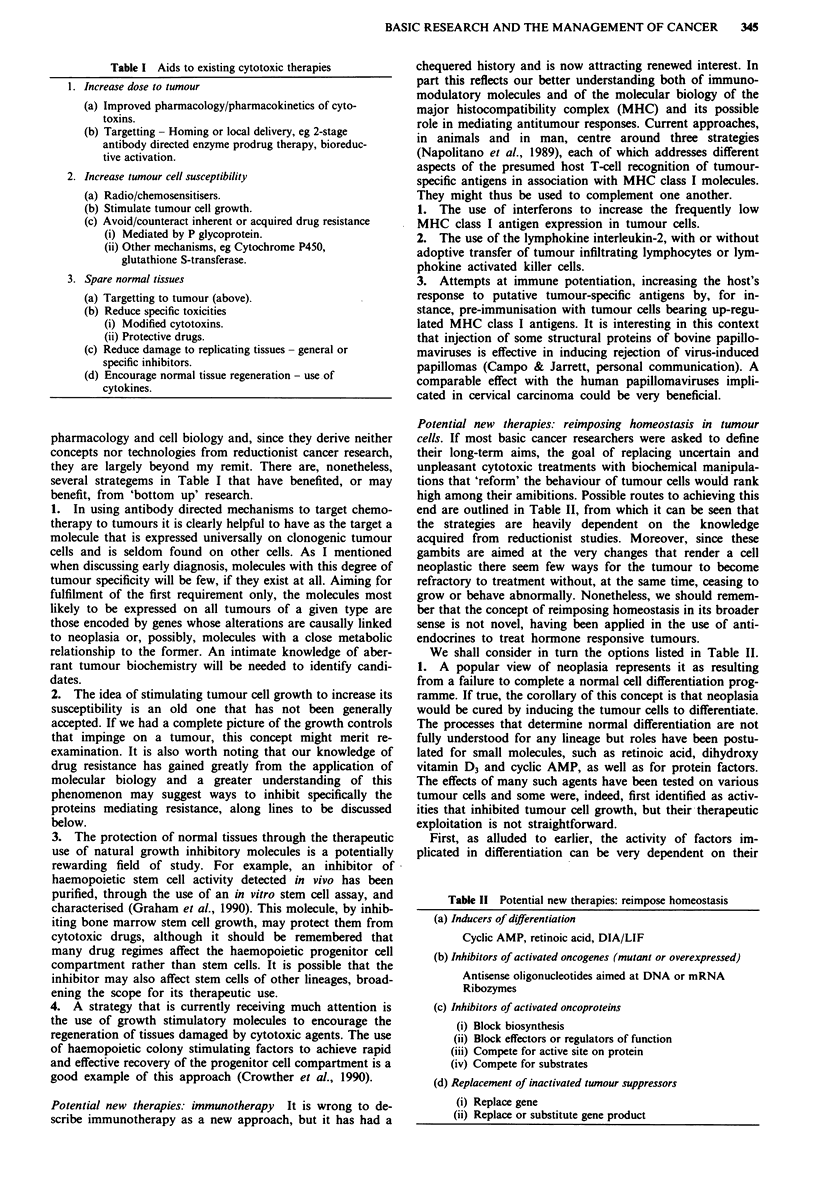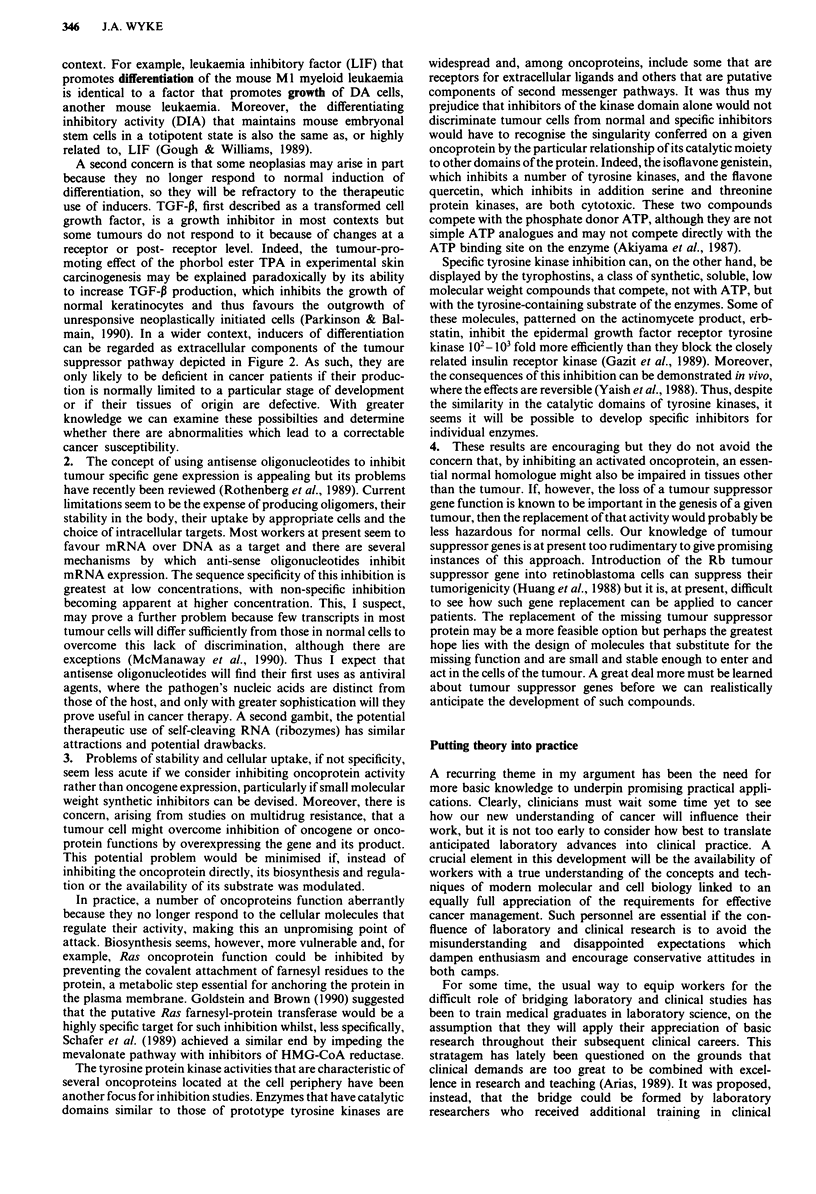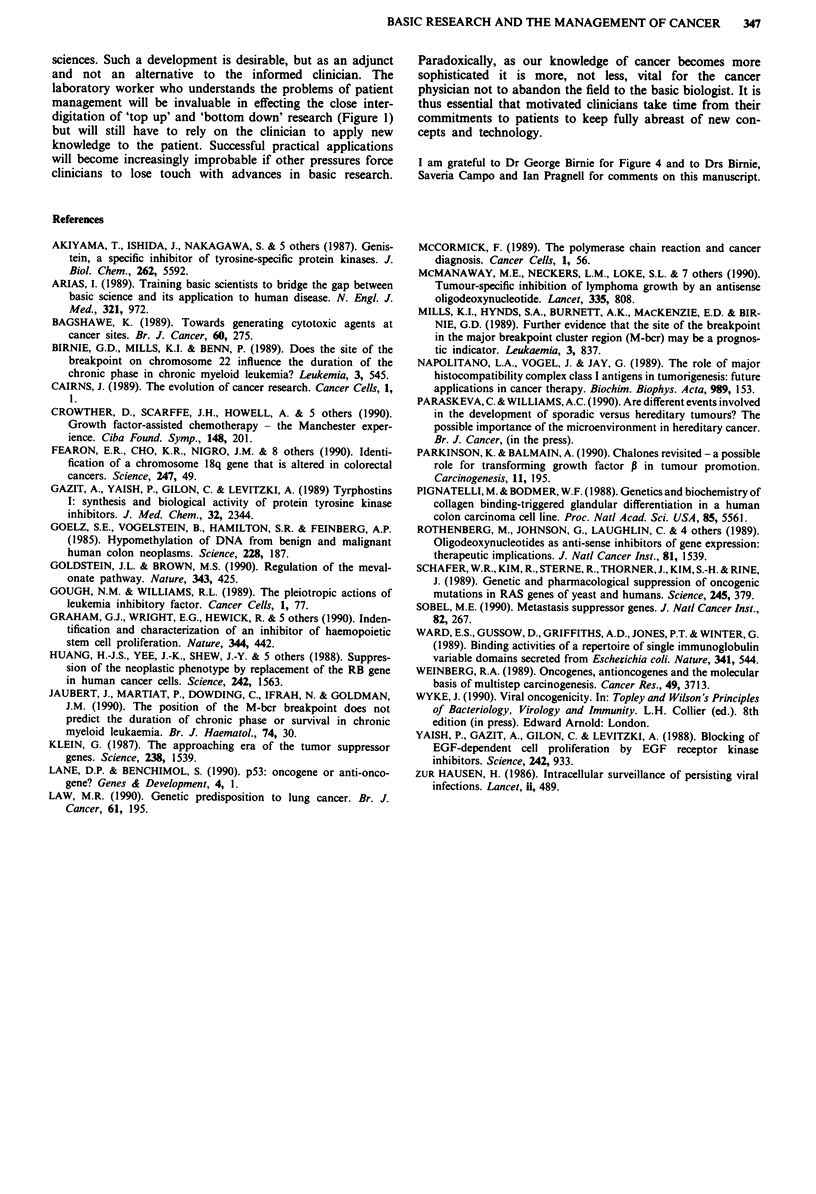# The second Bagshawe lecture. Matching basic research to the management of cancer: the view from the other side of the fence.

**DOI:** 10.1038/bjc.1990.294

**Published:** 1990-09

**Authors:** J. A. Wyke

**Affiliations:** CRC Beatson Laboratories, Beatson Institute for Cancer Research, Glasgow, UK.


Br. J. Cancer (1990), 62, 341–347 © Macmillan Press Ltd., 1990

### THE SECOND BAGSHAWE LECTURE*

### Matching basic research to the management of cancer: the view from the other side of the fence

J.A. Wyke

*CRC Beatson Laboratories, Beatson Institute for Cancer Research, Garscube Estate, Switchback Road, Bearsden, Glasgow G61 1BD, UK.*

In his inaugural lecture in this series, Professor Ken Bagshawe hoped that his successors 'will not shrink from controversy or unwheeling contemporary bandwagons' (Bagshawe, 1989). The Association of Cancer Physicians has now asked me to inspect, on their behalf, one of the biggest of all bandwagons, modern basic cancer research. You would not wish me to unwheel, even if I could, a vehicle with so many passengers, but you are concerned about its direction and its rate of progress, an impatience ironically acknowledged in Professor Bagshawe's original title for his lecture; 'Whilst waiting for the human genome to be mapped'. Like all mechanics, I am underneath this bandwagon looking up and, despite erratic steering and a lumbering forward motion, it seems quite capable of reaching its destination if carefully handled. I hope to persuade you of this by examining, firstly, our current understanding of cancer biology, secondly, the prospects this offers for better management and, finally, ways to encourage the translation of understanding into practice.

### The biologist's view of cancer

Like most long-standing endeavours, the present status of basic cancer research is only fully explicable by reference to its history. The original impetus to know more about cancer came from the problems of managing the cancer patient and laboratory investigations were intended, directly or indirectly, to aid this management. The knowledge gained from this 'top down' approach and, in particular, the finding that a single cancer cell could form a tumour in a recipient animal, led to the cancer cell supplanting the cancer patient as the unit of interest to many biologists. There developed the large body of 'bottom up' cancer research, that tackled problems generated at the cellular level and whose reductionist view was greatly abetted by the development of *in vitro* cell culture systems, particularly those exploiting tumour viruses and the power of classical molecular genetics (Figure 1).

This latter approach has proved enormously fruitful, leading in turn to the identification of viral genes that induce neoplasia, to the discovery of normal cell homologues (proto-oncogenes) for some of these viral oncogenes and to the implication of proto-oncogene mutations in many naturally-occurring cancers. These findings, in total, impressively validate the concept that somatic mutations in the neoplastic cell lineage underlie the altered growth and behaviour that typify cancer. Moreover, functions were assigned to many proto-oncogene products that seemingly explained their ability to perturb normal cell growth and behaviour. It has become axiomatic that genes whose alterations contribute to neoplasia encode products that play a role in the complex processes by which a cell perceives and responds to its environment. 'Oncoproteins' have variously been identified as molecules transmitting signals between cells, as the receptors for these ligands (either on the cell surface or inside the cell), as components of second messenger pathways or as nuclear proteins that mediate the response of the cell genome (Figure 2). We might also expect oncoproteins to contribute to events that follow from changes in genomic activity, but clear cut examples of changes in these 'effector' pathways are so far lacking, in contrast to the probable situation with tumour suppressor genes (see below, Fearon *et al.*, 1990; Pignatelli & Bodmer, 1988). The signalling pathways in which oncoproteins have been implicated have generally been those favouring cell multiplication at the expense of terminal differentiation (Figure 2) but it must be emphasised that in no case (with the possible exception of oncoproteins cognate with intracellular hormone receptors) do we understand in detail the circuits disrupted by oncogenic mutations.

The success of the reductionist approach warrants its continuation for as long as we can ask significant questions about the cancer cell and the issue of matching this research to the management of the patient is rightly irrelevant to its justification. However, over the past decade the molecular biologist has come to appreciate the complexity of neoplasia that has long been apparent to the pathologist and physician and the limitations of pure reductionism have been exposed. The viral oncogene paradigm and its conceptual descendants provide an inherent bias to the detection of **genetic** alterations that, **on their own**, confer on a normal cell **phenotypically dominant altered growth**. Attention has thus been diverted from, and is only recently returning to, the following crucial facets of neoplasia.

**Figure 1** The structure of cancer research, depicted as a pyramid whose apex is the desired solution to managing all or part of the problem. Questions initially posed by attempts at patient management stimulated both clinical research (top of pyramid) and 'top down' laboratory work (filled arrowheads). The latter soon led to broadly-based, self-justifying 'bottom up' investigations, based on the cancer cell (cross-hatched arrowheads). The two types of laboratory work were, until recently, generally distinguishable in concepts and techniques, but they are now merging as the principles of basic research are applied to problems posed by the patient and should soon impinge on clinical practice.

Received 20 April 1990.
*Presented at the 31st Annual Meeting of the BACR and 5th Annual Meeting of the ACP, 19–22 March 1990.

**Figure 2** Schematic of the pathways that control cell growth and differentiation. The proliferation-promoting pathway shows a signal (ligand) binding to a transmembrane receptor to generate a second message that leads, directly or indirectly, to a change in nuclear function. An example is the binding of epidermal growth factor to its receptor, the latter being a tyrosine protein kinase cognate with the *erb*-B oncogene. The differentiation-promoting pathway shows an example of a signal recognised by an intracellular receptor that can itself bind to DNA, an instance being steroid hormone receptors (in the diagram the receptor is nuclear but it may frequently be cytoplasmic). These examples are only two of many possible variations that can operate in either type of pathway. The pathways themselves are not necessarily distinct and opposing, as shown in this simple diagram, but may communicate and a single component (such as transforming growth factor-β or leukaemia inhibitory factor) can contribute to different pathways in different cell environments (from Wyke, 1990, with permission).

*Tumour evolution* The multistep character of tumour development was the first focus for molecular biologists grappling with the more complex aspects of neoplasia (Weinberg, 1989). Information on this point from the well-tried experimental systems retained, however, the bias to identifying what were then considered to be dominantly acting oncogenes.

*Tumour suppression* The concept that recessive (loss of function) mutations contribute as much, or more, to neoplasia than dominant (change of level or function) mutations is now a major bandwagon in its own right (Klein, 1987). The tumour suppressor genes that are the targets for such mutations were first highlighted in model systems but their recent prominence is due to studies on naturally occurring tumours in which, moreover, their mutations have been shown to contribute to the multistep evolution of cancer (Fearon *et al.*, 1990). These findings exemplify a significant trend in which the technologies of the reductionist approach are increasingly applied to problems of 'top down' cancer research to reveal concepts that were inaccessible to earlier experimental systems. These challenges stimulate, at the active interface between 'bottom up' and 'top down' studies, the development of more sophisticated models, such as transgenic animals bearing specific mutations in oncogenes or tumour suppressor genes.

There is growing evidence that tumour suppressor gene and oncogene functions occupy comparable niches in cellular physiology, with the former mediating processes that favour differentiation over cell multiplication (Figure 2). Furthermore, the perceived functional contrast between the two sets of genes is likely, for a variety of reasons, to be somewhat artificial. The fine-tuning of cell homeostasis will require extensive 'cross-talk' between pathways that promote or inhibit a given aspect of cell activity and natural parsimony may dictate that similar proteins have different functions in different contexts (the behaviour of transforming growth factor-β (TGF-β) is a good example of this, see below). Furthermore, the distinction between oncogene and tumour suppressor gene may be operational rather than absolute, and the same gene may be assigned to both categories, depending on the mutations it has suffered. The best example of this is the p53 gene (Lane & Benchimol, 1990) but since its behaviour is still incompletely understood it may be better to illustrate the point with a purely hypothetical case (Figure 3). Suppose a protein, A, modifies a substrate, B, the product B* being essential for a process of terminal differentiation (Figure 3(i)). Mutations, A′, that prevent A binding to B will be phenotypically recessive (Figure 3(ii)) but, if the wild type A allele is also mutated or lost, differentiation will be blocked and neoplasia may ensue, a characteristic scenario with tumour suppressor gene mutations. Other loss of function mutations, A″, that lead to a high affinity binding of B, but

**Figure 3** Three types of mutation that might be implicated in neoplasia. This hypothetical example postulates (i) a protein, A, whose normal function is to convert a target molecule, B, into a modified product, B*, that is required for differentiation. (ii) A mutant, A′, that cannot interact with B would be recessive, impeding differentiation only if the normal protein is lost. By this criterion A would be considered a typical tumour suppressor protein. (iii) If a mutant protein, A″, has lost its normal function but displays an abnormal affinity for B, this would have a dominant negative effect. It would be relieved if there is enough non-sequestrated B protein for conversion to B* by the wild type A product. (iv) A mutation that, for example, changes the location or regulation of active A might expose it to a target, related to B, but responsible for a proliferative stimulus that overrides the continuing signal to differentiate. The mutant protein, A‴, is thus equivalent to a dominantly acting oncoprotein.

no modification, could have a dominant negative effect, leading to a block in differentiation that is, however, partially relieved by the presence of wild type A product (Figure 3(iii)). Finally, Figure 3(iv) exemplifies one of several types of mutation whose effect would be dominant even in the presence of wild type A. In this case a mutant protein of altered location (A‴) binds to a local B-related substrate, C, that in modified form mediates a cell proliferative signal that may or may not preclude acquisition of differentiated characteristics.

*Changes in cell behaviour* Most genetic alterations that have been implicated so far in cancer have had effects on cell multiplication, yet it is the concomitant changes in cell behaviour that pose the greatest challenges in managing the disease. The interactions of tumour cells with adjacent normal cells (and, through signalling molecules, with distant normal cells), and the tumour cells' ability to invade locally, disseminate, attach and grow in ectopic locations and furnish themselves with a blood supply are all important phenomena for which our knowledge is rudimentary (Paraskeva & Williams, 1990; Sobel, 1990; Weinberg, 1989). In part this is because most of these facets of tumour biology are only properly examined in the whole organism, a difficult theatre of operations for the cell and molecular biologist. Here again we might hope that studies on natural tumours will provide useful correlations between genetic alterations and tumour behaviour that, in turn, will permit the design of appropriate model systems for further investigation.

*Non-genetic alterations* A central thesis in modern cancer research has been the importance of somatic (and sometimes germline) mutations in the tumour lineage. Without decrying the importance of mutation, it seems that normal metazoan development involves changes in cellular phenotype, usually without alterations in genotype, that are largely irreversible and thus embody the stability required to play a role in neoplasia. There is only sparse information on whether mechanisms that regulate gene activity in the long term, such as transitions in chromatin configuration and DNA methylation (Goelz *et al.*, 1985), have any significance for cancer causation. These are, indeed, difficult areas of study but I suspect that their relative neglect, like those of the other topics outlined above, stems from the undoubted success of mainstream reductionist studies.

To sum up, the success of the past 20 years of basic cancer research has taken us to a new level of understanding of cancer biology. For the first time we have a clear picture of what awaits discovery and, although our ignorance is daunting, some cancer biologists are already trying to rebuild the process in all its complexity even as their colleagues continue to disassemble it. It is undoubtedly early days to predict confidently how this growth in basic understanding will affect cancer patients but, armed with examples from the literature and the work of my colleagues, I will indicate where applications can be anticipated.

### Applying cancer biology to cancer management

Even incomplete basic knowledge expands the options for intervention in cancer (Cairns, 1989) and opportunities can be foreseen for improving management of all aspects of the problem.

*Cancer avoidance* Our discussion so far has been about mechanisms, rather than causes, of cancer and might be thought of little relevance to preventing the disease. However, studies on mechanism have provided information in several areas germane to avoidance.

Tumour viruses, which are risk factors in about 10–20% of human cancers (zur Hausen, 1986), have been a favourite tool of the basic biologists and our appreciation of their clinical significance has benefited greatly from this attention. Prophylactic vaccines, whose development required an intimate knowledge of the viruses, have been developed for oncogenic herpesviruses, papillomaviruses and retroviruses of animals and for hepatitis B virus of man. Vaccines against the other human viruses implicated in cancer, Epstein Barr virus and papilloma- and retroviruses, are at varying stages of research or development. We must bear in mind, however, that the rationale for developing such vaccines is not necessarily straightforward (Wyke, 1990). Viruses implicated in human cancer are generally widespread agents and neoplasia is characteristically associated with chronic infection, acquired early in life and accompanied by immune impairment and exposure to co-carcinogens. A significant risk of neoplasia may thus only exist for a subset of the population and there may be a lag of a generation before benefits accrue from immunisation. These factors mean that the benefits of vaccination must be weighed carefully against its costs and risks unless, as with hepatitis B, the virus poses a major non-neoplastic risk to health.

Consideration of viral carcinogenesis leads us to another aspect of cancer avoidance that may benefit from deeper biological understanding. Controlled breeding and intensive husbandry in the domestic fowl have shown that susceptibility to the commonest form of cancer is widespread and inherited in a dominant fashion. The reason has been known for 30 years. In domestication, the retroviruses of the avian sarcoma/leukosis complex are the predominant carcinogens and they can only infect birds that express cellular receptors for the viral envelope glycoproteins. Such clear-cut predisposition to neoplasia is only rarely seen in human populations that are generally outbred and exposed to varying environmental influences, but the possibility of more subtle and ubiquitous variations in inherited proneness to cancer is now receiving attention (for example, Law, 1990). If we understand the undoubtedly complex factors that determine such variations then we can tackle the even more difficult problem of deciding how to use such knowledge.

In the even longer term, there are two other prospects for cancer avoidance, and the fowl again provides an example of the first. If a strain of chickens is susceptible to virus-induced neoplasia because it expresses the cellular receptor for a common retrovirus type, then it is possible to abrogate this susceptibility by manipulation of the chicken genome. Classical breeding has achieved this in some instances but transgenesis now provides, in theory, two other options. Site specific recombination can be used to ablate the gene encoding the viral receptor or the receptor can be blocked by introducing into the bird a vector expressing the viral envelope glycoprotein. We do not know, however, the physiological role of the receptor molecule, so we are unable to predict the consequences of either its loss or permanent association with the viral glycoprotein ligand. Such uncertainties may only be resolved by studying the transgenic animal and thus practical, as well as ethical, considerations may largely limit this type of intervention to veterinary subjects.

The other long term prospect is the improved detection of environmental carcinogens. At present a battery of tests are used to predict carcinogenicity or mutagenicity but these do not examine the precise pathways by which carcinogens exert their effects. However, once the details of carcinogenesis are understood it may be possible to devise tests that predict more exactly the consequences of exposure to a given agent. This knowledge will help in deciding appropriate safety controls as well as in monitoring the effects of untoward exposure. In a broader context, and one that is even more difficult to predict, a greater understanding of mechanisms may help in elucidating the causes of those common cancers in which environmental influences are suspected but not defined. This should be a very important goal, but epidemiological successes do not necessarily lead to improved cancer management if their implications run counter to social and political trends. Political considerations also apply to the next aspect of management that will benefit from basic knowledge.

*Screening and early detection of cancer* I am not qualified to comment on the economic, social and psychological ramifications of detecting cancer at an earlier stage but, objectively, early detection may increase, and should never reduce, the patient's lifespan. However, although biologists may leave others to decide the desirability of screening in specific instances, they have to recognise that any potential screening strategies spawned by basic research should be simple, cheap, precise, easy to interpret and acceptable to the population to be screened.

These are severe and sometimes opposing constraints to place on the detection of a complex and subtle disease in which a series of genetic alterations lead to either qualitative or quantitative changes in gene products. Thus, simplicity, economy and, to a lesser extent, precision, are best satisfied by screening for gene products that invariably undergo tumour-specific qualitative changes that can be detected with high sensitivity, probably by immunological means. Acceptability would be enormously enhanced if these changes could be perceived in samples obtained by non-invasive means, presumably biasing this approach to tumours of the exocrine organs and the respiratory, alimentary and urogenital tracts where, fortunately, many important human cancers occur. It will not be easy to discover tumour cell products with these stringent characteristics.

In contrast, the most precise and easily interpreted changes are those to the DNA of tumour cells, but it has been difficult to see how genetic alterations to a small number of cells could be detected with high sensitivity and patient compliance. The rapid exploitation of the polymerase chain reaction (PCR) in the past few years looks set to solve the problem of sensitivity if not acceptability (McCormick, 1989). However, just as detailed knowledge of protein alterations are essential to devising immunological probes, so we must understand precisely the sites of mutations in tumours to design oligonucleotide primers for PCR. The inescapable conclusion is that more basic information is needed to underpin new screening strategies.

*Diagnosis and prognosis* Even without the development of new early detection methods, existing screens for carcinoma of the cervix and breast will produce false positives that need rapid resolution, adding to an important demand on expert diagnostic services. There is thus a clear incentive to ask whether our new knowledge of cancer will allow diagnosis to become easier and cheaper without sacrificing its accuracy. It is also important to know whether prognosis can be made more precise, particularly in predicting response to therapy and thus contributing more to the management of the disease. In addition there is a need for more effective monitoring of the establishment and maintenance of remission in patients already receiving treatment.

The approaches which basic research can bring to bear on these needs are similar to those which can be applied to early detection, notably antibody probes for tumour-specific proteins and nucleic acid probes for genetic alterations. However, applications in diagnosis and prognosis are greater and constraints are less. Indeed, although monitoring remission for incipient relapse shares many of the problems of early detection, it is relatively free of the need for simplicity, low cost and patient compliance and can, moreover, be tailored to individual patients. Thus, overall, this is the first facet of cancer management in which our new biological understanding is likely to have an impact. Monoclonal antibodies for immune histochemistry are readily used and may soon be supplemented by 'single domain antibodies', themselves a result of exploiting PCR technology (Ward *et al*., 1989). The detection of nucleic acid changes, be they characteristic RNA transcripts or genomic DNA altered by deletions, point mutations or translocation have formerly required techniques that were relatively complex for adoption by pathology laboratories. However, attempts to simplify their use by devising various *in situ* hybridisation techniques may themselves soon be supplanted by the widespread adoption of PCR.

At present, indeed, the limitations to applying molecular biology to diagnosis are not in the techniques but in our ability to interpret the results, and until our understanding is more profound that will restrict implications for cancer management. One example illustrates these points. The Philadelphia chromosomes are produced by translocations that fuse the 5′ end of the *phl* (*BCR*) gene on chromosome 22 to a decapitated c-*abl* gene from chromosome 9, the hybrid gene producing mRNA transcripts and protein products that are unique to chronic myeloid leukaemia (CML). Although the breakpoints on chromosome 22 in CML occur in a limited region (Figure 4a), they do show variations that modify the structure of the *phl-abl* hybrid gene and several groups have tried to correlate these variations with the clinical course of CML. Birnie *et al*. (1989) have discussed these studies and proposed, as a consensus, that breakpoints with a more 5′ location in *phl* are found more frequently in patients with a longer chronic phase to the disease (Figure 4b), although this conclusion remains in dispute (Jaubert *et al*., 1990). Clearly, more basic information is needed to decide whether and how chromosome 22 breakpoint positions influence CML prognosis and only then can it be determined whether this information will influence patient management.

*Aids to existing cytotoxic therapies* Cancer physicians are most excited by the scope basic understanding of cancer offers for new and improved treatments. Prospects for therapy are of two types; adjuncts to existing treatments or radically new approaches to treatment, the former probably entering clinical research and practice before the latter.

Some ways of improving existing cytotoxic chemotherapy or radiotherapy are listed in Table I. Most of the gambits mentioned evolved from 'top down' research in chemistry,

**Figure 4** **a**, The structure of various hybrid genes and hybrid transcripts between *phl* (*BCR*) (chromosome 22, open boxes) and c-*abl* (chromosome 9, filled boxes). Introns in the genes are shown by straight lines. The question mark indicates that the transcript depicted has yet to be found and, if it does exist, the coding region of the *abl* portion would be expected to be out of frame with the *phl* portion. The boundary between 5′ breakpoints (to the left) and 3′ (to the right) is arbitrarily located at about the middle of the intron between *phl* exons 14 and 15. **b**, Duration of chronic phase for Philadelphia chromosome positive CML patients with either 5′ or 3′ breakpoint (from Mills *et al*., 1989).

**Table I** Aids to existing cytotoxic therapies

1. *Increase dose to tumour*
   (a) Improved pharmacology/pharmacokinetics of cytotoxins.
   (b) Targetting – Homing or local delivery, eg 2-stage antibody directed enzyme prodrug therapy, bioreductive activation.
2. *Increase tumour cell susceptibility*
   (a) Radio/chemosensitisers.
   (b) Stimulate tumour cell growth.
   (c) Avoid/counteract inherent or acquired drug resistance
      (i) Mediated by P glycoprotein.
      (ii) Other mechanisms, eg Cytochrome P450, glutathione S-transferase.
3. *Spare normal tissues*
   (a) Targetting to tumour (above).
   (b) Reduce specific toxicities
      (i) Modified cytotoxins.
      (ii) Protective drugs.
   (c) Reduce damage to replicating tissues – general or specific inhibitors.
   (d) Encourage normal tissue regeneration – use of cytokines.

pharmacology and cell biology and, since they derive neither concepts nor technologies from reductionist cancer research, they are largely beyond my remit. There are, nonetheless, several strategems in Table I that have benefited, or may benefit, from 'bottom up' research.

**1.** In using antibody directed mechanisms to target chemotherapy to tumours it is clearly helpful to have as the target a molecule that is expressed universally on clonogenic tumour cells and is seldom found on other cells. As I mentioned when discussing early diagnosis, molecules with this degree of tumour specificity will be few, if they exist at all. Aiming for fulfilment of the first requirement only, the molecules most likely to be expressed on all tumours of a given type are those encoded by genes whose alterations are causally linked to neoplasia or, possibly, molecules with a close metabolic relationship to the former. An intimate knowledge of aberrant tumour biochemistry will be needed to identify candidates.

**2.** The idea of stimulating tumour cell growth to increase its susceptibility is an old one that has not been generally accepted. If we had a complete picture of the growth controls that impinge on a tumour, this concept might merit re-examination. It is also worth noting that our knowledge of drug resistance has gained greatly from the application of molecular biology and a greater understanding of this phenomenon may suggest ways to inhibit specifically the proteins mediating resistance, along lines to be discussed below.

**3.** The protection of normal tissues through the therapeutic use of natural growth inhibitory molecules is a potentially rewarding field of study. For example, an inhibitor of haemopoietic stem cell activity detected *in vivo* has been purified, through the use of an *in vitro* stem cell assay, and characterised (Graham *et al.*, 1990). This molecule, by inhibiting bone marrow stem cell growth, may protect them from cytotoxic drugs, although it should be remembered that many drug regimes affect the haemopoietic progenitor cell compartment rather than stem cells. It is possible that the inhibitor may also affect stem cells of other lineages, broadening the scope for its therapeutic use.

**4.** A strategy that is currently receiving much attention is the use of growth stimulatory molecules to encourage the regeneration of tissues damaged by cytotoxic agents. The use of haemopoietic colony stimulating factors to achieve rapid and effective recovery of the progenitor cell compartment is a good example of this approach (Crowther *et al.*, 1990).

*Potential new therapies: immunotherapy* It is wrong to describe immunotherapy as a new approach, but it has had a chequered history and is now attracting renewed interest. In part this reflects our better understanding both of immunomodulatory molecules and of the molecular biology of the major histocompatibility complex (MHC) and its possible role in mediating antitumour responses. Current approaches, in animals and in man, centre around three strategies (Napolitano *et al.*, 1989), each of which addresses different aspects of the presumed host T-cell recognition of tumour-specific antigens in association with MHC class I molecules. They might thus be used to complement one another.

**1.** The use of interferons to increase the frequently low MHC class I antigen expression in tumour cells.

**2.** The use of the lymphokine interleukin-2, with or without adoptive transfer of tumour infiltrating lymphocytes or lymphokine activated killer cells.

**3.** Attempts at immune potentiation, increasing the host's response to putative tumour-specific antigens by, for instance, pre-immunisation with tumour cells bearing up-regulated MHC class I antigens. It is interesting in this context that injection of some structural proteins of bovine papillomaviruses is effective in inducing rejection of virus-induced papillomas (Campo & Jarrett, personal communication). A comparable effect with the human papillomaviruses implicated in cervical carcinoma could be very beneficial.

*Potential new therapies: reimposing homeostasis in tumour cells.* If most basic cancer researchers were asked to define their long-term aims, the goal of replacing uncertain and unpleasant cytotoxic treatments with biochemical manipulations that 'reform' the behaviour of tumour cells would rank high among their amibitions. Possible routes to achieving this end are outlined in Table II, from which it can be seen that the strategies are heavily dependent on the knowledge acquired from reductionist studies. Moreover, since these gambits are aimed at the very changes that render a cell neoplastic there seem few ways for the tumour to become refractory to treatment without, at the same time, ceasing to grow or behave abnormally. Nonetheless, we should remember that the concept of reimposing homeostasis in its broader sense is not novel, having been applied in the use of anti-endocrines to treat hormone responsive tumours.

We shall consider in turn the options listed in Table II.

**1.** A popular view of neoplasia represents it as resulting from a failure to complete a normal cell differentiation programme. If true, the corollary of this concept is that neoplasia would be cured by inducing the tumour cells to differentiate. The processes that determine normal differentiation are not fully understood for any lineage but roles have been postulated for small molecules, such as retinoic acid, dihydroxy vitamin $D_3$ and cyclic AMP, as well as for protein factors. The effects of many such agents have been tested on various tumour cells and some were, indeed, first identified as activities that inhibited tumour cell growth, but their therapeutic exploitation is not straightforward.

First, as alluded to earlier, the activity of factors implicated in differentiation can be very dependent on their

**Table II** Potential new therapies: reimpose homeostasis

(a) *Inducers of differentiation*
   Cyclic AMP, retinoic acid, DIA/LIF

(b) *Inhibitors of activated oncogenes (mutant or overexpressed)*
   Antisense oligonucleotides aimed at DNA or mRNA
   Ribozymes

(c) *Inhibitors of activated oncoproteins*
   (i) Block biosynthesis
   (ii) Block effectors or regulators of function
   (iii) Compete for active site on protein
   (iv) Compete for substrates

(d) *Replacement of inactivated tumour suppressors*
   (i) Replace gene
   (ii) Replace or substitute gene product context. For example, leukaemia inhibitory factor (LIF) that promotes **differentiation** of the mouse M1 myeloid leukaemia is identical to a factor that promotes **growth** of DA cells, another mouse leukaemia. Moreover, the differentiating inhibitory activity (DIA) that maintains mouse embryonal stem cells in a totipotent state is also the same as, or highly related to, LIF (Gough & Williams, 1989).

A second concern is that some neoplasias may arise in part because they no longer respond to normal induction of differentiation, so they will be refractory to the therapeutic use of inducers. TGF-$\beta$, first described as a transformed cell growth factor, is a growth inhibitor in most contexts but some tumours do not respond to it because of changes at a receptor or post- receptor level. Indeed, the tumour-promoting effect of the phorbol ester TPA in experimental skin carcinogenesis may be explained paradoxically by its ability to increase TGF-$\beta$ production, which inhibits the growth of normal keratinocytes and thus favours the outgrowth of unresponsive neoplastically initiated cells (Parkinson & Balmain, 1990). In a wider context, inducers of differentiation can be regarded as extracellular components of the tumour suppressor pathway depicted in Figure 2. As such, they are only likely to be deficient in cancer patients if their production is normally limited to a particular stage of development or if their tissues of origin are defective. With greater knowledge we can examine these possibilties and determine whether there are abnormalities which lead to a correctable cancer susceptibility.

**2.** The concept of using antisense oligonucleotides to inhibit tumour specific gene expression is appealing but its problems have recently been reviewed (Rothenberg *et al.*, 1989). Current limitations seem to be the expense of producing oligomers, their stability in the body, their uptake by appropriate cells and the choice of intracellular targets. Most workers at present seem to favour mRNA over DNA as a target and there are several mechanisms by which anti-sense oligonucleotides inhibit mRNA expression. The sequence specificity of this inhibition is greatest at low concentrations, with non-specific inhibition becoming apparent at higher concentration. This, I suspect, may prove a further problem because few transcripts in most tumour cells will differ sufficiently from those in normal cells to overcome this lack of discrimination, although there are exceptions (McManaway *et al.*, 1990). Thus I expect that antisense oligonucleotides will find their first uses as antiviral agents, where the pathogen's nucleic acids are distinct from those of the host, and only with greater sophistication will they prove useful in cancer therapy. A second gambit, the potential therapeutic use of self-cleaving RNA (ribozymes) has similar attractions and potential drawbacks.

**3.** Problems of stability and cellular uptake, if not specificity, seem less acute if we consider inhibiting oncoprotein activity rather than oncogene expression, particularly if small molecular weight synthetic inhibitors can be devised. Moreover, there is concern, arising from studies on multidrug resistance, that a tumour cell might overcome inhibition of oncogene or oncoprotein functions by overexpressing the gene and its product. This potential problem would be minimised if, instead of inhibiting the oncoprotein directly, its biosynthesis and regulation or the availability of its substrate was modulated.

In practice, a number of oncoproteins function aberrantly because they no longer respond to the cellular molecules that regulate their activity, making this an unpromising point of attack. Biosynthesis seems, however, more vulnerable and, for example, *Ras* oncoprotein function could be inhibited by preventing the covalent attachment of farnesyl residues to the protein, a metabolic step essential for anchoring the protein in the plasma membrane. Goldstein and Brown (1990) suggested that the putative *Ras* farnesyl-protein transferase would be a highly specific target for such inhibition whilst, less specifically, Schafer *et al.* (1989) achieved a similar end by impeding the mevalonate pathway with inhibitors of HMG-CoA reductase.

The tyrosine protein kinase activities that are characteristic of several oncoproteins located at the cell periphery have been another focus for inhibition studies. Enzymes that have catalytic domains similar to those of prototype tyrosine kinases are widespread and, among oncoproteins, include some that are receptors for extracellular ligands and others that are putative components of second messenger pathways. It was thus my prejudice that inhibitors of the kinase domain alone would not discriminate tumour cells from normal and specific inhibitors would have to recognise the singularity conferred on a given oncoprotein by the particular relationship of its catalytic moiety to other domains of the protein. Indeed, the isoflavone genistein, which inhibits a number of tyrosine kinases, and the flavone quercetin, which inhibits in addition serine and threonine protein kinases, are both cytotoxic. These two compounds compete with the phosphate donor ATP, although they are not simple ATP analogues and may not compete directly with the ATP binding site on the enzyme (Akiyama *et al.*, 1987).

Specific tyrosine kinase inhibition can, on the other hand, be displayed by the tyrophostins, a class of synthetic, soluble, low molecular weight compounds that compete, not with ATP, but with the tyrosine-containing substrate of the enzymes. Some of these molecules, patterned on the actinomycete product, erbstatin, inhibit the epidermal growth factor receptor tyrosine kinase $10^2-10^3$ fold more efficiently than they block the closely related insulin receptor kinase (Gazit *et al.*, 1989). Moreover, the consequences of this inhibition can be demonstrated *in vivo*, where the effects are reversible (Yaish *et al.*, 1988). Thus, despite the similarity in the catalytic domains of tyrosine kinases, it seems it will be possible to develop specific inhibitors for individual enzymes.

**4.** These results are encouraging but they do not avoid the concern that, by inhibiting an activated oncoprotein, an essential normal homologue might also be impaired in tissues other than the tumour. If, however, the loss of a tumour suppressor gene function is known to be important in the genesis of a given tumour, then the replacement of that activity would probably be less hazardous for normal cells. Our knowledge of tumour suppressor genes is at present too rudimentary to give promising instances of this approach. Introduction of the Rb tumour suppressor gene into retinoblastoma cells can suppress their tumorigenicity (Huang *et al.*, 1988) but it is, at present, difficult to see how such gene replacement can be applied to cancer patients. The replacement of the missing tumour suppressor protein may be a more feasible option but perhaps the greatest hope lies with the design of molecules that substitute for the missing function and are small and stable enough to enter and act in the cells of the tumour. A great deal more must be learned about tumour suppressor genes before we can realistically anticipate the development of such compounds.

### Putting theory into practice

A recurring theme in my argument has been the need for more basic knowledge to underpin promising practical applications. Clearly, clinicians must wait some time yet to see how our new understanding of cancer will influence their work, but it is not too early to consider how best to translate anticipated laboratory advances into clinical practice. A crucial element in this development will be the availability of workers with a true understanding of the concepts and techniques of modern molecular and cell biology linked to an equally full appreciation of the requirements for effective cancer management. Such personnel are essential if the confluence of laboratory and clinical research is to avoid the misunderstanding and disappointed expectations which dampen enthusiasm and encourage conservative attitudes in both camps.

For some time, the usual way to equip workers for the difficult role of bridging laboratory and clinical studies has been to train medical graduates in laboratory science, on the assumption that they will apply their appreciation of basic research throughout their subsequent clinical careers. This stratagem has lately been questioned on the grounds that clinical demands are too great to be combined with excellence in research and teaching (Arias, 1989). It was proposed, instead, that the bridge could be formed by laboratory researchers who received additional training in clinical sciences. Such a development is desirable, but as an adjunct and not an alternative to the informed clinician. The laboratory worker who understands the problems of patient management will be invaluable in effecting the close interdigitation of 'top up' and 'bottom down' research (Figure 1) but will still have to rely on the clinician to apply new knowledge to the patient. Successful practical applications will become increasingly improbable if other pressures force clinicians to lose touch with advances in basic research. Paradoxically, as our knowledge of cancer becomes more sophisticated it is more, not less, vital for the cancer physician not to abandon the field to the basic biologist. It is thus essential that motivated clinicians take time from their commitments to patients to keep fully abreast of new concepts and technology.

I am grateful to Dr George Birnie for Figure 4 and to Drs Birnie, Saveria Campo and Ian Pragnell for comments on this manuscript.

**References**

AKIYAMA, T., ISHIDA, J., NAKAGAWA, S. & 5 others (1987). Genistein, a specific inhibitor of tyrosine-specific protein kinases. *J. Biol. Chem.*, **262**, 5592.

ARIAS, I. (1989). Training basic scientists to bridge the gap between basic science and its application to human disease. *N. Engl. J. Med.*, **321**, 972.

BAGSHAWE, K. (1989). Towards generating cytotoxic agents at cancer sites. *Br. J. Cancer*, **60**, 275.

BIRNIE, G.D., MILLS, K.I. & BENN, P. (1989). Does the site of the breakpoint on chromosome 22 influence the duration of the chronic phase in chronic myeloid leukemia? *Leukemia*, **3**, 545.

CAIRNS, J. (1989). The evolution of cancer research. *Cancer Cells*, **1**, 1.

CROWTHER, D., SCARFFE, J.H., HOWELL, A. & 5 others (1990). Growth factor-assisted chemotherapy – the Manchester experience. *Ciba Found. Symp.*, **148**, 201.

FEARON, E.R., CHO, K.R., NIGRO, J.M. & 8 others (1990). Identification of a chromosome 18q gene that is altered in colorectal cancers. *Science*, **247**, 49.

GAZIT, A., YAISH, P., GILON, C. & LEVITZKI, A. (1989) Tyrphostins I: synthesis and biological activity of protein tyrosine kinase inhibitors. *J. Med. Chem.*, **32**, 2344.

GOELZ, S.E., VOGELSTEIN, B., HAMILTON, S.R. & FEINBERG, A.P. (1985). Hypomethylation of DNA from benign and malignant human colon neoplasms. *Science*, **228**, 187.

GOLDSTEIN, J.L. & BROWN, M.S. (1990). Regulation of the mevalonate pathway. *Nature*, **343**, 425.

GOUGH, N.M. & WILLIAMS, R.L. (1989). The pleiotropic actions of leukemia inhibitory factor. *Cancer Cells*, **1**, 77.

GRAHAM, G.J., WRIGHT, E.G., HEWICK, R. & 5 others (1990). Indentification and characterization of an inhibitor of haemopoietic stem cell proliferation. *Nature*, **344**, 442.

HUANG, H.-J.S., YEE, J.-K., SHEW, J.-Y. & 5 others (1988). Suppression of the neoplastic phenotype by replacement of the RB gene in human cancer cells. *Science*, **242**, 1563.

JAUBERT, J., MARTIAT, P., DOWDING, C., IFRAH, N. & GOLDMAN, J.M. (1990). The position of the M-bcr breakpoint does not predict the duration of chronic phase or survival in chronic myeloid leukaemia. *Br. J. Haematol.*, **74**, 30.

KLEIN, G. (1987). The approaching era of the tumor suppressor genes. *Science*, **238**, 1539.

LANE, D.P. & BENCHIMOL, S. (1990). p53: oncogene or anti-oncogene? *Genes & Development*, **4**, 1.

LAW, M.R. (1990). Genetic predisposition to lung cancer. *Br. J. Cancer*, **61**, 195.

MCCORMICK, F. (1989). The polymerase chain reaction and cancer diagnosis. *Cancer Cells*, **1**, 56.

MCMANAWAY, M.E., NECKERS, L.M., LOKE, S.L. & 7 others (1990). Tumour-specific inhibition of lymphoma growth by an antisense oligodeoxynucleotide. *Lancet*, **335**, 808.

MILLS, K.I., HYNDS, S.A., BURNETT, A.K., MACKENZIE, E.D. & BIRNIE, G.D. (1989). Further evidence that the site of the breakpoint in the major breakpoint cluster region (M-bcr) may be a prognostic indicator. *Leukaemia*, **3**, 837.

NAPOLITANO, L.A., VOGEL, J. & JAY, G. (1989). The role of major histocompatibility complex class I antigens in tumorigenesis: future applications in cancer therapy. *Biochim. Biophys. Acta*, **989**, 153.

PARASKEVA, C. & WILLIAMS, A.C. (1990). Are different events involved in the development of sporadic versus hereditary tumours? The possible importance of the microenvironment in hereditary cancer. *Br. J. Cancer*, (in the press).

PARKINSON, K. & BALMAIN, A. (1990). Chalones revisited – a possible role for transforming growth factor $\beta$ in tumour promotion. *Carcinogenesis*, **11**, 195.

PIGNATELLI, M. & BODMER, W.F. (1988). Genetics and biochemistry of collagen binding-triggered glandular differentiation in a human colon carcinoma cell line. *Proc. Natl Acad. Sci. USA*, **85**, 5561.

ROTHENBERG, M., JOHNSON, G., LAUGHLIN, C. & 4 others (1989). Oligodeoxynucleotides as anti-sense inhibitors of gene expression: therapeutic implications. *J. Natl Cancer Inst.*, **81**, 1539.

SCHAFER, W.R., KIM, R., STERNE, R., THORNER, J., KIM, S.-H. & RINE, J. (1989). Genetic and pharmacological suppression of oncogenic mutations in RAS genes of yeast and humans. *Science*, **245**, 379.

SOBEL, M.E. (1990). Metastasis suppressor genes. *J. Natl Cancer Inst.*, **82**, 267.

WARD, E.S., GUSSOW, D., GRIFFITHS, A.D., JONES, P.T. & WINTER, G. (1989). Binding activities of a repertoire of single immunoglobulin variable domains secreted from *Escherichia coli*. *Nature*, **341**, 544.

WEINBERG, R.A. (1989). Oncogenes, antioncogenes and the molecular basis of multistep carcinogenesis. *Cancer Res.*, **49**, 3713.

WYKE, J. (1990). Viral oncogenicity. In: *Topley and Wilson's Principles of Bacteriology, Virology and Immunity*. L.H. Collier (ed.). 8th edition (in press). Edward Arnold: London.

YAISH, P., GAZIT, A., GILON, C. & LEVITZKI, A. (1988). Blocking of EGF-dependent cell proliferation by EGF receptor kinase inhibitors. *Science*, **242**, 933.

ZUR HAUSEN, H. (1986). Intracellular surveillance of persisting viral infections. *Lancet*, **ii**, 489.